# Molecular mechanism implicated in Pemetrexed-induced apoptosis in human melanoma cells

**DOI:** 10.1186/1476-4598-11-25

**Published:** 2012-04-26

**Authors:** Aitziber Buqué, Jangi Sh Muhialdin, Alberto Muñoz, Begoña Calvo, Sergio Carrera, Unai Aresti, Aintzane Sancho, Itziar Rubio, Guillermo López-Vivanco

**Affiliations:** 1 Medical Oncology Research Group, Hospital Universitario Cruces, Barakaldo, Bizkaia, Spain; 2 Medical Oncology Department, Hospital Universitario Cruces, Barakaldo, Bizkaia, Spain

**Keywords:** Pemetrexed, MTA, Antifolates, DNA damage, Apoptosis, Melanoma

## Abstract

**Background:**

Metastatic melanoma is a lethal skin cancer and its incidence is rising every year. It represents a challenge for oncologist, as the current treatment options are non-curative in the majority of cases; therefore, the effort to find and/or develop novel compounds is mandatory. Pemetrexed (Alimta®, MTA) is a multitarget antifolate that inhibits folate-dependent enzymes: thymidylate synthase, dihydrofolate reductase and glycinamide ribonucleotide formyltransferase, required for *de novo* synthesis of nucleotides for DNA replication. It is currently used in the treatment of mesothelioma and non-small cell lung cancer (NSCLC), and has shown clinical activity in other tumors such as breast, colorectal, bladder, cervical, gastric and pancreatic cancer. However, its effect in human melanoma has not been studied yet.

**Results:**

In the current work we studied the effect of MTA on four human melanoma cell lines A375, Hs294T, HT144 and MeWo and in two NSCLC cell lines H1299 and Calu-3. We have found that MTA induces DNA damage, S-phase cell cycle arrest, and caspase- dependent and –independent apoptosis. We show that an increment of the intracellular reactive oxygen species (ROS) and p53 is required for MTA-induced cytotoxicity by utilizing N-Acetyl-L-Cysteine (NAC) to blockage of ROS and p53-defective H1299 NSCLC cell line. Pretreatment of melanoma cells with NAC significantly decreased the DNA damage, p53 up-regulation and cytotoxic effect of MTA. MTA was able to induce p53 expression leading to up-regulation of p53-dependent genes Mcl-1 and PIDD, followed by a postranscriptional regulation of Mcl-1 improving apoptosis.

**Conclusions:**

We found that MTA induced DNA damage and mitochondrial-mediated apoptosis in human melanoma cells in vitro and that the associated apoptosis was both caspase-dependent and –independent and p53-mediated. Our data suggest that MTA may be of therapeutic relevance for the future treatment of human malignant melanoma.

## Background

Melanoma is one of the less common types of skin cancers, but nevertheless causes the majority of skin cancer-related deaths. In 2010, more than 60,000 new cases of invasive melanoma were diagnosed in the United States, with more than 7,000 recorded related deaths; according to the 2011 WHO report, about 48,000 melanoma-related deaths occur worldwide per year [1,2]. The standard treatment for localized melanoma is surgical excision, but melanoma that has spread to distant sites is rarely curable with standard therapy. The most active chemotherapy for metastatic melanoma is Dacarbazine (DTIC). However, this drug causes cell shrinkage in only 8-20% of treated patients, the response is partial and it lasts for a mere 4 to 6 months. Immunotherapy with interleukin-2 (IL-2) or interferon (IFN) at high-doses has been found to increase relapse-free survival (RFS) and overall survival (OS), but the high-dose regimes have important side effects. Therefore the identification of new agents that selectively kill melanoma cells is of primary interest.

Pemetrexed (MTA) is a novel multitarget antifolate with promising clinical activity. At present, it is a first line therapy against mesothelioma and non small cell lung cancer (NSCLC). However, its role in other malignancies and particularly in malignant melanoma has not yet been studied. MTA targets the folate-dependent enzymes thymidylate synthetase (TS), dihydrofolate reductase (DHFR) and glycinamide ribonucleotide formyltransferase (GARFT), all of which are involved in the *de novo* biosynthesis of purines and pyrimidines, thereby inducing an imbalance in the nucleotide pool and consequent DNA damage [3]; nevertheless, the precise details of its mechanism of action remain unknown.

One of the many effector mechanisms involved in the control and regulation of apoptosis is the cellular redox status which is determined by the balance between the rates of production and breakdown of reactive oxygen and/or nitrogen species (ROS; RNS). The mitochondrion is the major intracellular source of ROS and [4] low levels of ROS are maintained in healthy cells due to the activity of various antioxidant systems. However, the tumor cell response to death stimuli has been shown to be a function of the cellular redox status and compounds which induce a significant increase in intracellular H_2_O_2_ are known to facilitate death execution by promoting DNA damage and/or genomic instability [5,6]. Following intracellular stress stimuli in response to DNA damage, p53 becomes activated and induces expression of several genes promoting cell cycle arrest and apoptosis [7].

In the present study we have evaluated the cytotoxic effects of MTA on different human melanoma cell lines. We found that MTA induces caspase-dependent and -independent apoptosis in human melanoma cells through intracellular ROS accumulation, which in turn promotes DNA damage and consequent p53-regulated gene expression changes. These findings indicate that MTA may have important novel therapeutic characteristics for the treatment of human metastatic melanoma.

## Methods

### Chemicals and reagents

Dulbecco’s Modified Eagle’s Medium (DMEM), Eagle’s Minimum Essential Medium (EMEM), RPMI 1640, sodium pyruvate, sodium bicarbonate, L-glutamine, streptomycin/penicillin antibiotic solution and fetal bovine serum (FBS) were all obtained from Cambrex (Cambrex Bio Science, Verviers, Belgium). Culture flasks, 96-, 24- and 6-well plates and centrifuge tubes were from Corning Incorporated (NY, USA). The XTT viability assay kit was purchased from Roche Molecular Biochemicals, and MTA was purchased from Lilly (Lilly France S.A.S, Fegersheim, France). Propidium iodide (PI), dimethylsulfoxide (DMSO), Triton X-100, bovine serum albumen (BSA), N-Acetyl-L-Cysteine (NAC), polyclonal rabbit anti cleaved-caspase-3 antibody and anti-actin antibody were purchased from Sigma Chemical Co. (St. Louis, MO, USA). Fluorescent labelled goat anti-rabbit IgG secondary antibody (Alexa Fluor®) and oxidation-sensitive fluorescent 2′,7′-dichlorodihydro-fluoresceindiacetate reagent (DCFH-DA) were from Molecular Probes (Europe BV, Leiden, The Netherlands). Mouse anti-cyt c monoclonal antibody, caspase colorimetric protease assay kit, caspase-2 (Z-VDVAD-FMK), -3 (Z-DEVD-FMK), -8 (Z-IETD-FMK), -9 (Z-LEHD-FMK), -10 (Z-AEVD-FMK) inhibitors and general caspase inhibitors (Z-VAD-FMK) were purchased from Biovision (Biovision Research Products, Mountain View, USA). Donkey anti-goat secondary antibody was from Santa Cruz Biotechnology, Inc. (Santa Cruz, CA, USA). HRP-labeled goat anti-rabbit IgGs and goat anti-mouse IgGs were purchased from Pierce Biotechnology Inc. (Meridian Rd., Rockford, USA). Acrylamide and bis-acrylamide solutions and Precision Plus Protein Standards were obtained from Bio-Rad Laboratories (Hercules, CA, USA).

### Cells and culture conditions

A375, Hs294T, HT144, MeWo, Calu-3 and H1299 cell lines were obtained from the American Type Culture Collection, ATCC (Rockville, Maryland). Primary mouse embryo fibroblasts (PMEF) were obtained from Millipore Corporation, MA, USA. Human melanoma cell lines A375, HT144 and Hs294T were maintained in cell culture medium supplemented with 10% FBS as described by ATCC. MeWo melanoma cell line and Calu-3 lung adenocarcinoma cell lines were maintained in minimum essential medium (Eagle) with 2 mM L-glutamine and Earle’s BSS adjusted to contain 1.5 g/L sodium bicarbonate, 0.1 mM non-essential amino acids and 1.0 mM sodium pyruvate and 10% FBS as described by ATCC. The H1299 lung carcinoma cell line was maintained in RPMI 1640 medium with 2 mM L-glutamine adjusted to contain 1.5 g/L sodium bicarbonate, 4.5 g/L glucose, 10 mM HEPES, and 1.0 mM sodium pyruvate and 10% FBS as described by ATCC.

Employed melanoma cell lines have different p53 genetic status as shown in Table 1.

**Table 1:** p53 genotype of employed cell lines

	**A375**	**Hs294T**	**HT144**	**MeWo**	**Calu-3**	**H1299**
TP53	wt	wt	wt	c.772G>A	c.711G>T	100% del.
				c.949C>T		

According to COSMIC database from Sanger institute ( http://www.sanger.ac.uk/genetics/CGP/cosmic) and “The TP53 web site” ( http://p53.free.fr/Database/Cancer_cell_lines/p53_cell_lines.html) the p53 status varies among the employed cell lines. A375, Hs294T and HT144 melanoma cell lines show a wt p53 while MeWo and Calu-3 have puntual mutations that seem to do not compromise its functional activity. On the other hand, H1299 does not show any p53 activity due to its 100% homozygous deletion.

MTA was dissolved in phosphate buffered saline (PBS) at 2 mg/ml and stored at −80°C until required. Cells were incubated for various periods of time with different concentrations of MTA (0.17-10 μM).

To verify the effect of ROS on MTA-induced cell death, ROS production was blocked by adding 10 mM N-acetyl-cysteine (NAC) 1 h before exposure to MTA.

### Cell proliferation and viability assays

Human cells were seeded into flat-bottomed 96-well microtiter plates at a density of 10^4^ cells per well in 100 μl of culture medium and allowed to attach to the wells overnight. Cells were treated with different concentrations of MTA (0–10 μM) for 24–72 h. Controls without MTA exposure were included in each experiment. Cell proliferation was determined by the colorimetric XTT viability assay [8]. The concentration of MTA required to inhibit viability by 50% (IC_50_) was determined using GraphPad Prism software (La Jolla, CA 92037, USA). All experiments were performed in triplicate, and the presented data represent the mean of three independent experiments.

### Clonogenic assay (colony-forming assay)

To determine the effect of MTA on cellular colony formation, 10^4^ cells per well were seeded in 6 well plates and allowed to adhere overnight. The cells were treated with 0.84 μM MTA for 24 h. The culture medium was subsequently replaced with MTA-free culture medium and the cells were incubated for 10 more days. The colonies were fixed with ice-cold methanol, stained with crystal violet, and viewed under a Nikon microscope (Nikon Eclipse TE2000-E); images were processed using NIS-Elements AR 2.31 software. All experiments were performed in triplicate, and the presented data represent the mean of three independent experiments.

### Morphological assays of apoptosis

#### Nuclear staining and fluorescent microscopy

Morphological evaluation of the nucleus before and after MTA exposure was performed by nuclear staining with PI as described previously [8]. Cells were viewed and analysed using a confocal microscope (Olympus FV 500), and images were obtained by sequential acquisition to avoid overlap.

#### Electron microscopy

For transmission electron microscopy (TEM), adherent cells were washed twice with 0.1 M cacodylate buffer, detached using a cell scraper, and mixed with cells floating in media. Cells were then centrifuged at 625 g for 7 min, and pellets were prefixed in 2.5% glutaraldehyde in 0.1 M cacodylate buffer for 1 h, followed by post fixation in 1% osmium tetraoxide (OsO4) in 0.2 M cacodylate buffer. Cells were subsequently dehydrated in an ascending graded ethanol series, embedded in resin, and polymerized for 48 h at 60 º C. Ultrathin sections were stained with uranyl acetate and examined using a Philips EM 208 electron microscope (Philips Electronic Instruments, Eindhoven, Netherlands).

### Cell cycle analysis

To quantify hypodiploid cells and analyze the cell cycle distribution of MTA-treated and untreated melanoma cells, these were subjected to flow cytometry analysis as described previously [8]. Analysis was performed on at least 10,000 cells using a Cytomics FC500 MPL (Beckman Coulter).

In transient knockdown of p53 transcriptional activity, cell cultures were pretreated with 30 μM of pifithrin-alpha (PFT-alpha) during 4 h. Subsequently, cell cultures were exposed to 0.17 and 0.82 μM MTA during 24 and 48 h. Finally, cells were stained and subjected to flow Cytometry.

### Mitochondrial membrane potential assay

The mitochondrial membrane potential (∆Ψm) of MTA-treated and untreated cells was analysed using DIOC6 as previously described [9]. For all conditions, 10,000 cells were analysed using a Cytomics FC 500MPL (Beckman Coulter).

### Quantification of reactive oxygen species (ROS)

To measure ROS production of cells before and after MTA exposure, 20 μM DCFH-DA was used as described previously [10]. For all conditions, 10,000 cells were analysed using a Cytomics FC 500MPL (Beckman Coulter).

### Comet assay

The comet assay (also known as single cell electrophoresis) was performed as described previously [8]. Digital images were obtained using a Nikon Eclipse TE2000-E fluorescence microscope.

### SDS-PAGE and immunoblotting

MTA-treated and untreated human melanoma cells were harvested by trypsinization. Cytosol and mitochondrial extracts were obtained by digitonin fractionation as previously described [11]; total cell lysate was prepared by resuspending pelleted cells in ice cold lysis buffer (1% Igepal, 1% Triton X-100, 20 mM Tris pH 7.3, 140 mM NaCl, 1 mM EDTA and water) containing protease and phosphatase inhibitors (3% protease inhibitor cocktail, 10 mM NaF, 10 mM NaPPi, 1 mM Sodium Vanadate, 1 mM PMSF) followed by 30 min incubation on ice. Aliquots of lysate were dissolved in Laemmli sample buffer, resolved by SDS-PAGE and transferred to PVDF membrane with the iBlot® Gel Transfer Device as described by the manufacturer. The following antibodies were used for immunoblotting: anti-cyt c (0.2 μg/ml), anti-actin (1:200) from Sigma, anti-Mcl1 (25 μg/ml) from R&D, anti-AIF (1:200) and anti-p53 (1:100) from SCBT, anti-PUMA (1:500) from ThermoScientific primary antibodies, and horseradish peroxidase (HRP)-conjugated secondary antibodies (1:5,000) both from ThermoScientific.

Images were acquired with the image analyzer G:Box (Syngene) and densitometry was carried out with the software GeneTools (Syngene).

### Immunocytochemistry

Cells were fixed in ice cold 70% ethanol for 30 min at 4°C before blocking with 1% BSA (v/v) for 1 h at room temperature. Primary antibodies diluted in 1% BSA buffer were added for overnight incubation at 4°C: anti-cleaved caspase-3 (1:500) and anti-cyt c antibody (2 μg/ml). After cells were washed, they were incubated for 1 h at room temperature in the corresponding labeled secondary antibodies. Nuclei were stained with Hoechst (1 μg/ml). Cells were viewed and analysed with a confocal microscope (Olympus FV 500) and the images were obtained by sequential acquisition to avoid overlap.

### Caspase activity assay

Caspase-2, -3, -8, -9 and −10 activities were measured in cell extracts (200 μg), using a colorimetric assay kit following the manufacturer’s instructions. Assays were performed in triplicate on 96-well plates using a microtiter plate reader at 405 nm. Fold increase in caspase activity was measured as caspase activity of MTA-treated cells divided by the corresponding activity in control cells.

Caspase inhibitors were used to confirm the dependency of apoptosis on caspase activation. In brief, cells were pre-treated with 25 μM caspases-2, -3, -8, -9, -10 and general caspase inhibitors for one hour, followed by treatment with MTA. Proliferation was subsequently evaluated with an XTT proliferation kit as described above.

### Real time RT-PCR

The level of expression of genes of interest was measured in each sample by one step real-time PCR using human validated TaqMan Gene Expression Assays (Ref. Hs01034253_m1 (P53), Hs00248075_m1 (BBC3), Hs00388035_m1 (LRDD) and Hs00172036_m1 (Mcl-1) from Applied Biosystems) with the Human RPLP0 (large ribosomal protein) Taqman Assay as the endogenous control. Three replicates were run for each sample in 96-well plates and 200 ng RNA were used for each reaction.

Assays were carried out in a 7900 HT Real Time PCR System (Applied Biosystems, Foster City, CA, USA) and analyses were done by the ΔΔCt method using the RQ manager software.

### Gene silencing assays

Melanoma cells were seeded in 12-well plates with corresponding medium without antibiotics overnight. The medium was replaced with fresh medium without serum and antibiotics. Silencing was performed using Lipofectamine and OptiMEM® following the instructions provided by the manufacturer. Briefly, 60 nM of commercially available p53 siRNA (Ambion) or negative control was mixed with 50 μl OptiMEM® and also, lipofectamine was mixed with 50 μl OptiMEM®. Both combinations were mixed and incubated for 20 min at 37°C. Finally the cells in each well were then transfected with this mixture. After 6 h, serum was added to a final concentration of 10%. 24 h later, cell culture medium was replaced with MTA-containing medium.

RNA and proteins were extracted using NucleoSpin miRNA Nucleic Acid and Protein Pufification (ref: 740971 from Macherey-Nagel)

### Statistical analysis

The statistical significance of differences between sample means was determined using the Student *t*-test; p values of < 0.05 were considered to be statistically significant.

## Results

### MTA induced apoptosis in human melanoma cells

**Figure 1:**
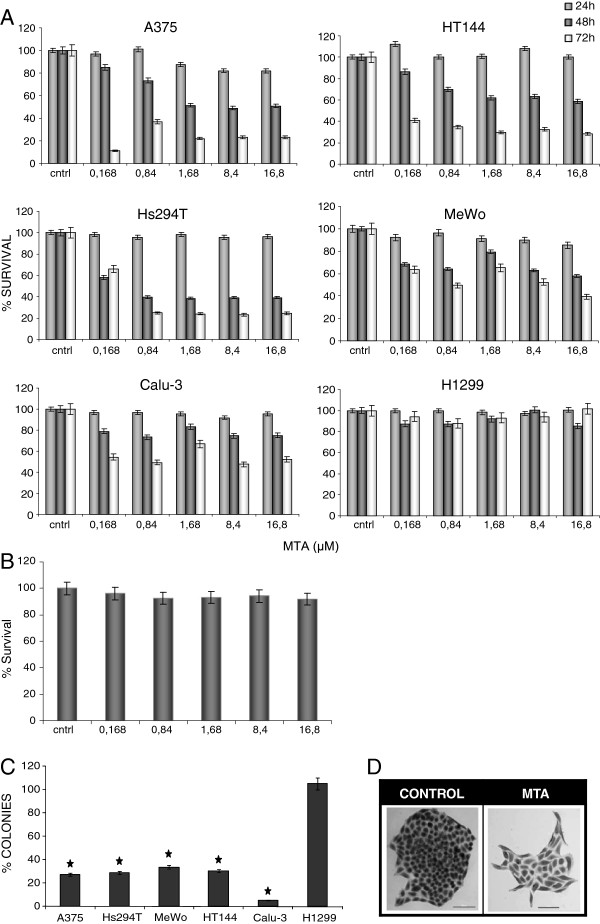
**Effects of MTA on human melanoma cell viability** (**A**) A375, Hs294T, HT144 and MeWo human melanoma cells were treated with different doses of MTA for 24, 48 and 72 h and cell viability was assessed by mean of the XTT viability assay. MTA significantly reduced cell viability in all melanoma cell lines and in Calu-3 while there was no significant effect on H1299 (a human NSCLC lacking p53 expression). Presented values are mean of at least 2 independent experiments. The IC50 values for MTA in A375, Hs294T, HT144 and MeWo human melanoma cells were 0.27, 0.41, 0.32 and 0.99 μM, respectively and 10 μM for the Calu-3 NSCLC line. Data represent mean ± SD of six determinations in three separate experiments for each cell line. (**B**) Effect of MTA on mouse embryonic fibroblast (MEF). Cells were exposed to the indicated MTA doses for 24, 48 and 72 h (only results of 72 h are shown). There was no significant cytotoxic effect on MEF cell line. Data represent mean ± SD of six determinations in two separate experiments. (**C**) Effect of MTA on the colony-forming ability of human melanoma cells and NSCLC cells. Cells were exposed to 0.84 μM MTA for 24 h followed by incubation in MTA-free culture medium for 10 days before performance of crystal violet staining and counting of colonies. MTA significantly reduced colony-forming ability in human melanoma cells and in Calu-3 cells, but not in the H1299 cell line, corroborating results obtained in the viability assay. *p < 0.05 (Student *t*-test). (**D**) Colony-forming ability: details of A375 cell line formed colonies growing in MTA-free medium (control) and in MTA-containing medium. This micrographs show that not only the amount of colonies buy also the morphology of formed colonies changed which are composed for significantly less number of cells.

We assessed the effect of MTA on cell proliferation using the XTT method on four human melanoma cell lines, on two types of NSCLC which constitute a positive control and on MEF cells to evaluate MTA cytotoxicity on non-tumoral cells. Cell viability decreased in a dose- and time-dependent manner in response to MTA, in all the studied human melanoma cell lines and in the positive control cell line Calu-3, but not in MEF or H1299, which lacks p53 expression due to a homozygous partial deletion (Figure 1 A and B).

Results of the clonogenic assay were consistent with the XTT data; MTA showed a negative effect on the colony formation ability of the A375, HT144, MeWo and Hs294T melanoma cell lines, but not on H1299 (Figure 1C and D).

**Figure 2:**
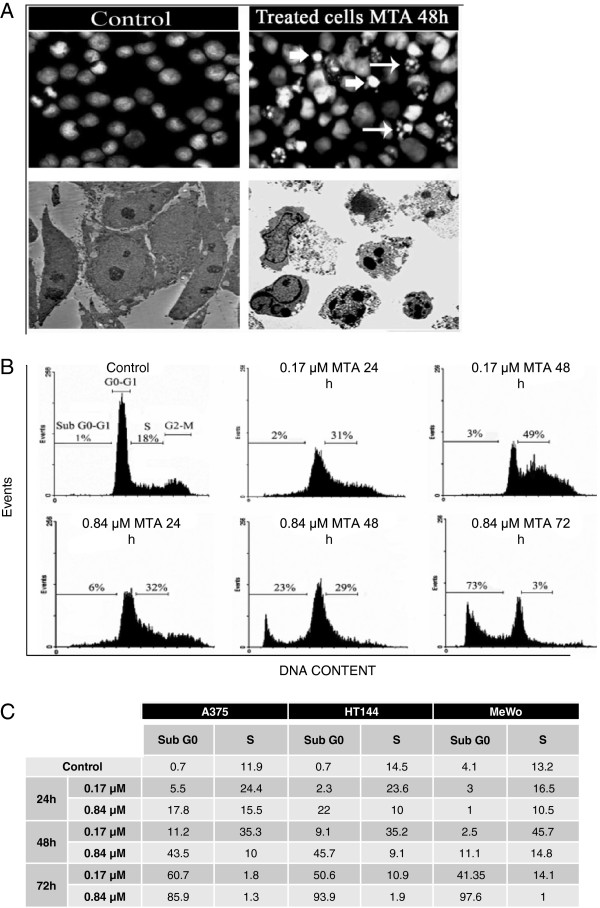
**MTA induced apoptosis in human melanoma cell lines** (**A**) The nature of cell death was identified by studying morphological changes which were apparent via immunocytochemistry and electron microscopy. Top: A375 representative fluorescence microscopy of nuclei stained with PI before and after MTA exposure. All fields were photographed at a magnification of 40 X. Bottom: A375 representative TEM images of cells before and after MTA exposure. Both techniques reveal that MTA exposure induced morphological changes such as DNA fragmentation and marginalization (arrows), which are characteristic of apoptosis. (B, C) Quantification of the extent of apoptosis was carried out by cell cycle analysis. Cells lines were treated with two different concentrations of MTA for 24, 48 and 72 h and analysed by flow cytometry. The results illustrated in (**B**) for Hs294T line are representative and the results for all cell lines are summarized in the table (**C**). Results are expressed as a percentage of the cell population in the subGo-G1 and S phases of three independent measurements. Cultures treated with MTA showed a time- and dose-dependent increase in their percentage of hypodiploid cells, with a modification of cell percentages in the S-phase of the cell cycle.

Morphological changes in the MTA-treated human melanoma cells were studied by fluorescent microscopy of cells stained with PI and by transmission electron microscopy, to evaluated ultrastructural characteristics. These studies revealed changes typical of apoptosis, such as nuclear condensation, fragmentation and margination and the dispersal of the nuclear fragments into the cytoplasm (Figure 2A).

Subsequently, we quantified apoptosis by flow cytometric analysis of the cell cycle. This analysis revealed that 0.84 μM MTA exposure increased the percentage of hypodiploid cells in subG0/G1 after 24, 48 and 72 h, respectively (Figure 2 B and C). Furthermore, exposure of cells to 0.17 μM MTA led to a transient arrest at the S phase, increasing cell population at this stage after 24 h and 48 h of treatment, respectively.

### Caspases participated in MTA-mediated apoptosis

**Figure 3:**
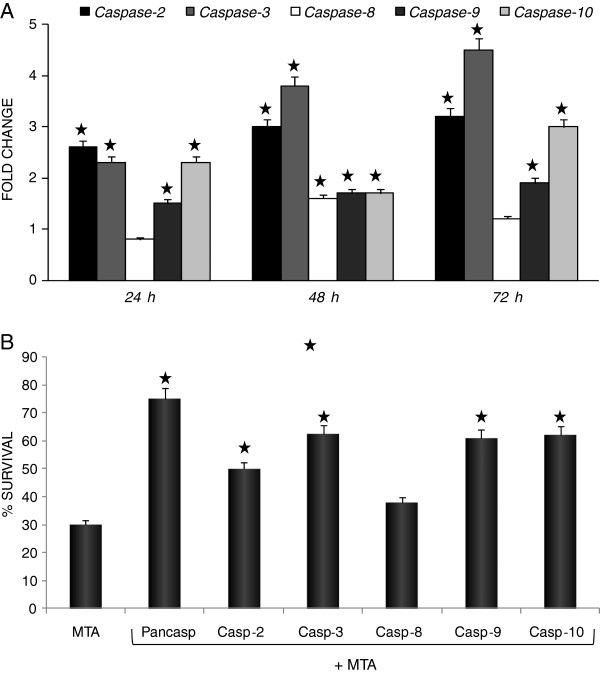
**MTA induced caspase-dependent apoptosis.** (**A**) Kinetics of caspase cascade activation. Induction of apoptosis in melanoma cells by MTA was accompanied by activation of caspases-2, -3, -8, -9 and −10. Hs294T cells were treated with 0.84 μM MTA for the indicated time points. Total cell lysates were obtained according to the manufacturer’s instructions. Assays were performed in triplicate in 96-well plates. Caspase activity results are represented as a fold change of the control, comparing obtained results (ORmta) with the activity obtained for MTA-untreated cells (ORcontrol) by computing ORmta/ORcontrol. Caspase-2 and −10 activities were enhanced after only 24 h, pointing to their role as inititor caspases. The activity of caspase-3 and −9 also increased significantly after 24 h MTA exposure, confirming their executioner role in this process. After 48 h MTA treatment, caspase-8 activity had also significantly increased, in addition to further increases in caspase-2 and −3 activities. Data represent mean ± SD of three determinations from three separate experiments. (**B**) Hs294T cells were pretreated with or without caspase inhibitors for 1 h and then challenged with 0.84 μM MTA for 72 h. Cell viability was assessed using the XTT assay. Blockade with the pancaspase inhibitor (z-VAD-FMK) inhibited significantly but not totally the MTA effect. The most effective caspase inhibitors were those which inhibited caspase-2 (z-VDVAD-FMK), caspase-3 (z-DEVD-FMK), caspase-9 (z-LEHD-FMK) and caspase-10 (z-AEVD-FMK) which confirmed the role of these caspases in mediating the effects due to MTA. The caspase-8 inhibitor (z-IETD-FMK) did not significantly reverse the MTA effect. These results are representative of three independent experiments. Similar results were obtained for all melanoma cell lines.

As caspases are the major mediators of apoptosis, we assessed the activity of caspases-2, -3, -8, -9, and −10 in human MTA-treated and untreated cells using a caspase colorimetric assay. MTA exposure for 24 h induced activation of initiator caspases −2 and −10, together with executioner caspases −3 and −9, with maximal activation being reached after 72 h of treatment (Figure 3A).

To clarify the role of the individual caspases in MTA-induced apoptosis in melanoma cells, we performed caspase-blocking experiments. Pre-treatment of Hs294T cells with a pan-caspase inhibitor, or with inhibitors to caspase-2, -3, -9 or −10, significantly inhibited MTA-induced apoptosis (Figure 3B). (Similar profiles were observed with the other human melanoma cell lines; not shown). These results indicate that this apoptotic process in human melanoma cells is mostly caspase-dependent, but do not rule out the possibility that caspase-independent factors may also participate in this process.

### Involvement of p53

**Figure 4:**
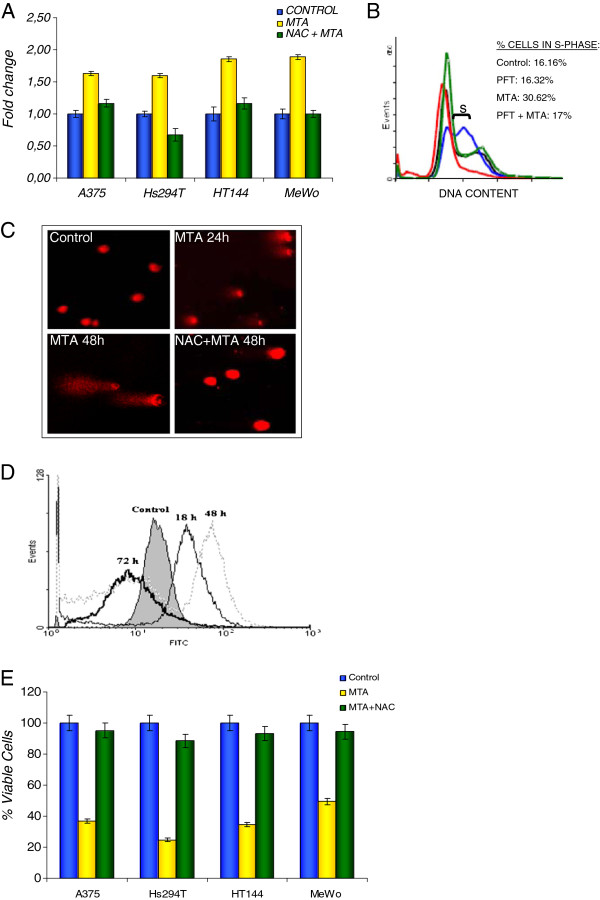
**MTA upregulated p53 expression, via DNA damage and intracellular ROS accumulation** (**A**) p53 expression was assessed by RT-PCR after 24 h treatment with 0.84 μM MTA. Raw data was extracted by the ΔΔCt method and expression results are represented as fold change relative to the expression of not exposed cells (control). MTA induced an increment of p53 expression comparing with controls. This increment was reduced when intracellular ROS levels were blocked with 10 mM NAC. These results are the mean of three independent experiments. (**B**) The role of p53 in cell cycle progression was verified by HT144 cell cycle distribution analysis by flow cytometry after inhibition of p53 transcriptional activity by PFT. Results are represented as the percentage of cells in S-phase. MTA-untreated cells (control) are represented in black, PFT-only treated cells in green, 0.17 μM MTA-only treated cells in blue and cells treated with both PFT and 0.17 μM MTA in red. MTA-induced cell cycle arrest at S-phase was avoided when inhibiting p53 activity. (**C**) DNA damage was analysed by Comet assay. Cells were exposed to 0.84 μM MTA for 24 or 48 h, after which double/single strand breaks were apparent. Less damage was observed when intracellular ROS levels were inhibited with 10 mM NAC. Illustrated fields are representative; magnification, 40 x. (**D**) MTA increased the level of ROS production. Intracellular ROS levels were measured by flow cytometry. Cells were exposed to 0.84 μM MTA for the indicated times before incubation with DCFH-DA for 60 min at 37°C. A significant increase in ROS production was apparent after 18 h in all melanoma cell lines. The graph illustrates the behaviour of the A375 cell line. (**E**) Cells were pretreated with 10 mM NAC before 0.84 μM MTA exposure. MTA-induced cytotoxicity was found to be inhibited in all employed melanoma cell lines when ROS was blocked. Results are expressed as the mean of three independent experiments, each one performed in triplicate.

It is well know that p53 plays a very important role in apoptotic cell death and development. Since we found that MTA did not induce any cytotoxic effect on the H1299 cell line, which lacks p53 expression, we evaluated the effect of MTA on p53 gene expression by RT-PCR in the four human melanoma cell lines and found that it led to an increase in p53 gene and protein expression (Figure 4A and Figure 6C). Given the strong role of p53 in regulating cell cycle arrest and preventing cell cycle replication during intra-S-phase checkpoint activation, we examined the cell cycle progression in HT144 cells after p53 activity inhibition by PFT-alpha. We observed a reduced number of cells at s-phase after p53 inhibition, suggesting a p53-regulated cell cycle arrest. (Figure 4B).

**Figure 5:**
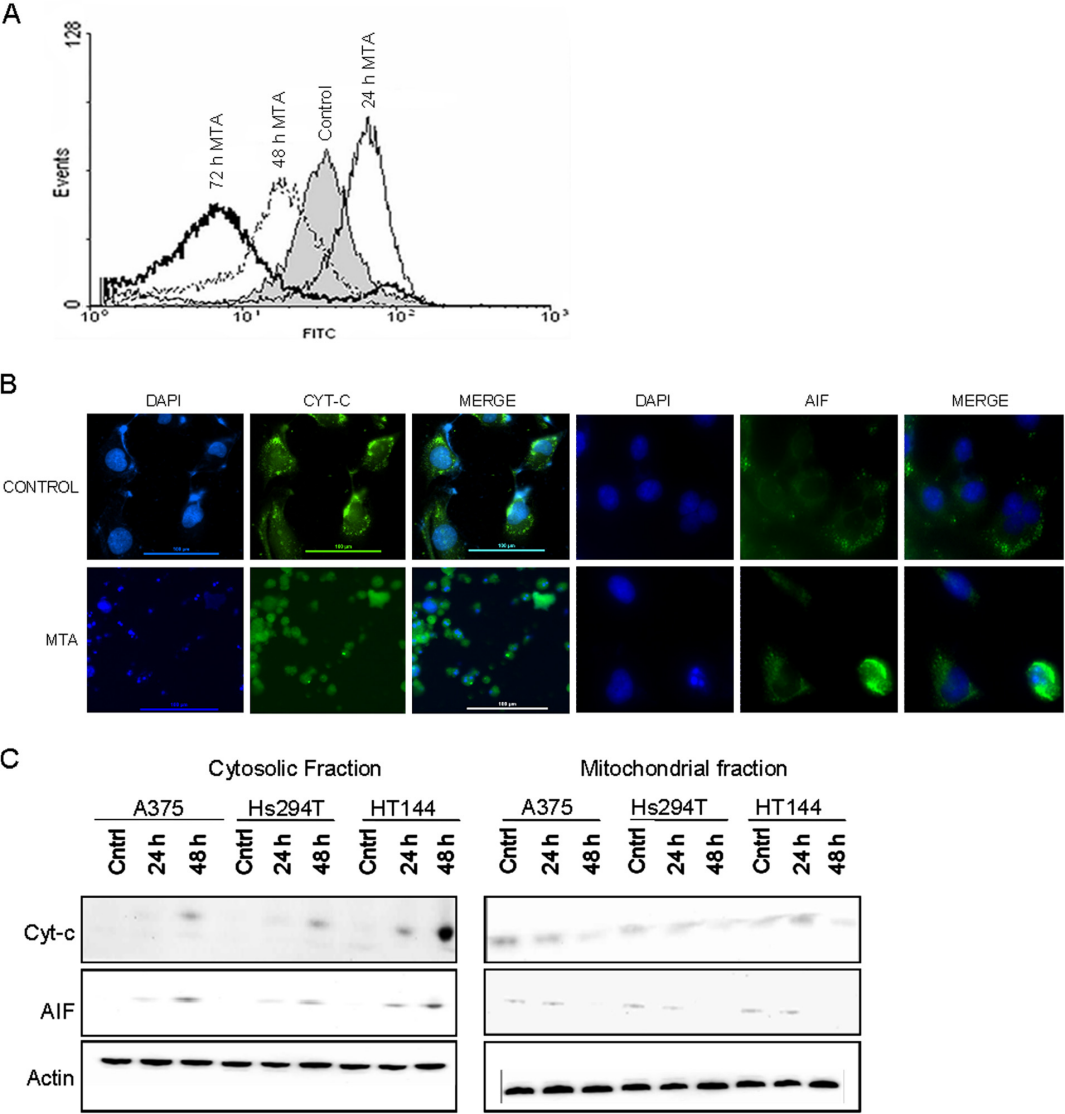
**Apoptosis effectors genes.** (**A**) p53 siRNA transfected and non- transfected cells were treated with MTA (0.84 μM) for 48 h before RNA extraction. Later, the expression of p53, and of the p53-related genes PUMA (BBC3), PIDD (LRDD) and Mcl-1 were analysed by RT-PCR. Results are represented in a heatmap showing the fold change of expression in three human melanoma cell lines, where upregulation is shown in red and downregulation in blue as shown on the rule on the right. We found that MTA induced the upregulation of PUMA, PIDD and Mcl-1 mRNA in all cell lines. However, when silencing p53 by siRNA, p53 was downregulated and there was a decreased upregulation of PIDD and Mcl-1 expression comparing with non-p53 silenced cells, pointing to a role of p53 in their regulation. PUMA expression did not change when p53 was silenced comparing with its expression in non-silenced cells, pointing to a p53-independent regulation. (**B**) Immunoblotting of cell lysates from cells treated with 0.84 μM MTA for the indicated time periods using antibodies specific for the indicated proteins. No changes in PUMA protein levels were observed and Mcl-1 protein appeared to fall after MTA treatment, suggesting that MTA may also exert its effect via postranscriptional modifications. (**C**) Immunoblotting of cell lysates from A375 cells with or without transiently silenced p53 by siRNA treated with 0.84 μM MTA for 48 h. On top, MTA induced the increment of p53 protein and around a 60% p53 reduction was silenced by siRNA assay. Middle, MTA induced a p53-dependent downregulation of Mcl-1 protein. Under each immunoblot ratios are shown, which the result of the normalisation of the raw volume of each sample with the corresponding actin’s value and the subsequent relativisation to the control. The blots were stripped and reprobed with actin which served as loading control.

To asses the hypothesis that MTA induces p53 expression due to DNA damage, we performed the Comet assay on MTA-treated and untreated cells and found significant DNA strand breaks after early MTA exposure (Figure 4C).

Tumoral cells, after exposure to chemotherapeutic agents typically respond by increasing intracellular ROS levels [5] via mitochondria. This increment can lead to DNA damage and apoptosis. We measured the intracellular ROS levels after MTA exposure and found that ROS levels significantly increased after 18 h of treatment, with maximum levels being reached after 48 h (Figure 4D: results from the HT144 cell line are representative of all melanoma cell lines). Next, we pretreated melanoma cells with 10 mM NAC 1 h prior to MTA exposure to reduce ROS accumulation and assessed viability, DNA damage and p53 expression. We found that NAC reversed cytotoxicity and reduced DNA damage and p53 expression (Figure 4 A, C, E).

### MTA activated a mitochondrial apoptosis pathway

The fact that MTA increased intracellular ROS levels suggested that MTA might involve changes in mitochondrial function. Thus, we analysed the mitochondrial membrane potential (∆Ψm) by flow cytometry. We found early mitochondrial membrane hyperpolarization in the A375 cell line, which has been interpreted by some authors as an early sign of apoptosis [12-14], followed by a later ∆Ψm depolarization, pointing to loss of mitochondrial membrane integrity (Figure 5A). Similar results were obtained with HT144, Hs294T, MeWo and Calu-3 cells (data not shown).

**Figure 6:**
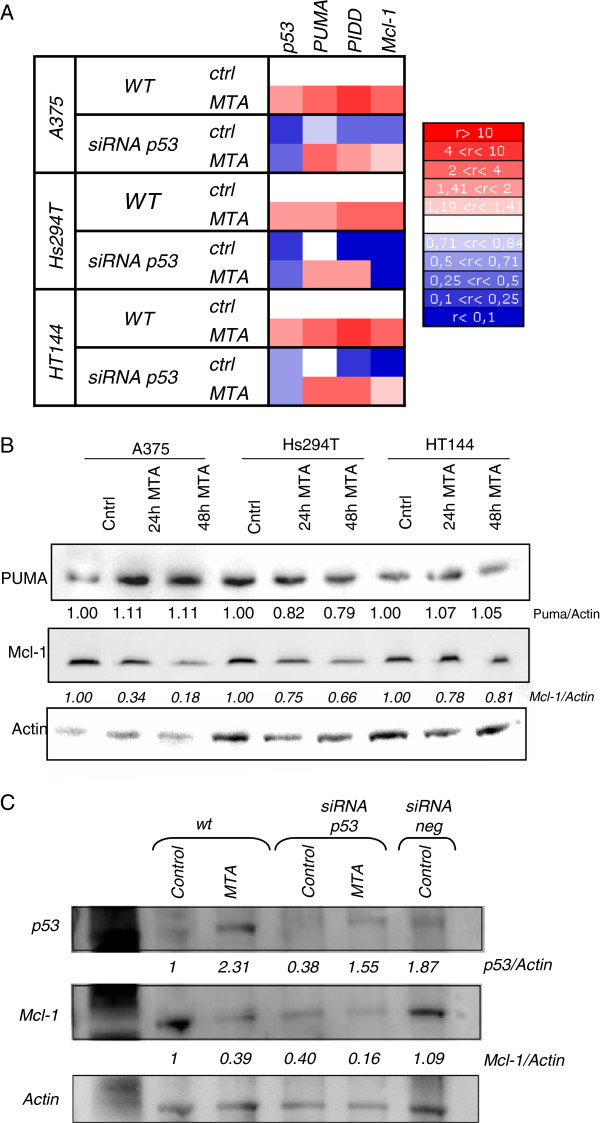
**MTA-induced apoptosis involved mitochondria** (**A**) MTA induced mitochondrial membrane damage in human melanoma cells as indicated by ∆Ψm collapse. Cells were incubated with 0.84 μM MTA for the indicated times. Subsequently, the mitochondrial membrane potential was measured by staining the cellls with 40 nM DiOC6. In the histogram, the curve shifted to the right, which indicates retention of the dye in the mitochondria (an early feature of apoptosis), followed by shifting of the curve to the left after 48 h due to collapse of the mitochondrial membrane potential. This figure is representative of 3 independent experiments. Similar values were obtained for the 4 melanoma cell lines and Calu-3 (data not shown). (**B**) MTA induced the release of cyt c and AIF from mitochondria. Cells were treated with 0.84 μM MTA for 48 h before immunocytochemistry. In untreated cells, cyt-c and AIF (green) exhibited a punctuate pattern which corresponds to the mitochondrial localization of these proteins. After 48 h of MTA exposure cyt-c and AIF immunoreactivity consisted of a diffuse pattern, indicative of their localization in the cytosol after the released from the mitochondria. This diffuse pattern of immunoreactivity was seen in cells which presented a fragmented nucleus (blue), indicative of apoptosis. (**C**) Immunoblotting of cell lysates from cytosolic and mitochondrial extracts of cells treated with 0.84 μM MTA for the indicated time periods using antibodies specific for the indicated proteins. The blots were stripped and reprobed with actin which served as loading control. Cyt c and AIF levels can be seen to decrease in the mitochondrial fraction while concomitantly increasing in the cytosolic fractions of MTA-treated cells.

To further corroborate the role of mitochondria in MTA-induced apoptosis, we analysed the release of the mitochondrial proteins cyt-c and AIF, to the cytosol. Confocal microscopic analysis of untreated cells showed that cyt c and AIF displayed a peripheral punctuate immunolabeling pattern consistent with mitochondrial staining. Addition of MTA caused redistribution of cyt c and AIF to the cytosol, as shown by a more diffuse, faint staining (Figure 5B). Western blot analysis further confirmed that upon treatment with MTA, the levels of cyt c and AIF in the mitochondrial fraction were reduced, in contrast to their levels in the cytosol which increased significantly (Figure 5C).

### Apoptosis-effectors genes

In order to analyze changes in expression of other possible apoptosis-effector genes, we analysed the behaviour of three p53-related genes which have been linked with the malignant transformation of melanocytes and with resistance to chemotherapy: BBC3 (PUMA), LRDD (PIDD) and Mcl-1 (Figure 6A). Expression of target genes was performed by real-time RT-PCR and raw data processed by the ΔΔCt method using human RPLPO gene as internal calibrator and MTA-unexposed cells as reference. We found that PUMA, PIDD and Mcl-1 genes were upregulated upon MTA treatment. Taken into account that these genes are highly regulated by p53 and in order to confirm the involvement of p53 in this MTA-related death, we performed a transient silencing of p53 by siRNA. Real-time RT-PCR assays resulted in a significant reduction of PIDD and Mcl-1 mRNA levels when p53 was silenced, suggesting an important role for p53 in both PIDD and Mcl-1 regulation; in contrast, the expression of PUMA appeared to be p53-independent given that p53 silencing did not cause any significant change, suggesting an alternative regulation (Figure 6A).

Despite the increased mRNA levels obtained by real-time RT-PCR, analysis of PUMA protein by western blot showed invariable levels after MTA treatment (Figure 6B). In the same way, Mcl-1 protein analysis by western blot showed a decrease of Mcl-1 upon MTA treatment, contrary to the increment observed at mRNA level. This decrement on Mcl-1 protein, as well as the gene expression, seemed to be p53-dependent (Figure 6C).

## Discussion

The results of the present study indicate that MTA is an active anti-melanoma agent in vitro. Treatment of human melanoma cells with MTA induced DNA damage, cell cycle arrest and caspase- and mitochondrial-mediated apoptosis. Induction of apoptosis is currently employed as an end point by which the efficacy of new therapeutic agents can be assessed in preclinical studies. Our results suggest that the efficacy of MTA in the treatment of human melanoma should be assessed by clinical trials, particularly because MTA is already in use for the treatment of other malignant diseases. Moreover, MTA is a well tolerated agent with acceptable toxicity if folic acid and vitamin B12 are prescribed with it [15,16].

Various in vitro and in vivo studies have examined the effects of MTA on various human tumour cells. Most of these studies have revealed that MTA, either alone or in combination with other drugs, can inhibit cell growth or induce a cytotoxic effect on tumour cells, such as breast, pancreas, lung and colon cancer cells [17]. In vivo, MTA has been administered in combination with other drugs such as platinum, gemcitabine and other antimetabolites and antitubulin agents, using EMT-mammary carcinoma, HCT116 colon cancer and H460 NSCLC xenografts in nude mice [18]. Importantly, MTA is currently used for the treatment of mesothelioma and non-small cell lung cancer, and has demonstrated clinical activity, either alone or in combination with the platinum compounds vinoralbine and gemcitabine, in a broad array of other solid tumors [15]. However, to our knowledge, no studies have been conducted on the effect of MTA on human melanoma, either in vitro or in vivo.

The current treatment options for patients with metastatic melanoma are limited and noncurative in the majority of cases. According to the American Joint Committee on Cancer, the usual outcome for patients with advanced metastatic melanoma (stage IV) remains bleak, with a median survival of six to ten months and less than 5% of patients surviving for more than five years [19-21]. Therefore, the identification of novel compounds and new strategies for more efficient treatment of this fatal disease is warranted. The testing of available compounds with known effects in other cancer therapies is a good starting point. Most of the therapeutic strategies used against cancer, such as immunotherapy, chemotherapy and irradiation, involve the induction of apoptosis in cancer cells [22]. Thus, one of the most important approaches for developing future cancer therapies is to understand the mechanisms by which successful therapies induce apoptosis.

Previous studies on the mechanism of action of MTA have shown that it inhibits cell proliferation and induces apoptosis in the cells of solid tumors [17,23] . In the current work, we studied the effect of MTA on four human melanoma cell lines. In addition, we examined MTA effects on two NSCLC cell lines (H1299 and Calu-3), which can be considered to be positive controls, since MTA is already used to treat this type of tumour. Our results revealed that MTA activates caspase cascades, and treatment with pan-caspase inhibitors significantly prevented MTA-induced apoptosis in human melanoma cells. This inhibition was not complete, indicating that other caspase-independent pathways may also participate in this cell death process.

Two pieces of evidence support the role of caspase-2 and −10 as key factors in initiating the MTA-mediated apoptotic process: first, these are the first caspases to be activated upstream of caspase-3 and −9 activation, and second, specific caspase-2 and −10 inhibitors effectively prevented MTA-induced apoptosis. The exact mechanism of caspase-2 and −10 activation is still unclear. Some reports have shown that caspase-2 and caspase-10 are initiator caspases related to the death receptor pathway [24,25]. In contrast, our results suggest that caspase-2 and −10 activation occurs upstream of caspase-8 activation and independent of the cell death receptors, since caspase-8 activation followed activation of caspase-2 and caspase-10, and the caspase-8 inhibitor was unable to significantly prevent cell death induced by MTA. In accordance with our results, other groups have reported that caspase-8 can be activated downstream of caspase-3 activation and independent of the death receptor pathway [26]. Recently, it was reported that caspase-2 and −10 can be recruited and activated by DNA damage [8,27]. To address this, we examined the genotoxic effect of MTA in human melanoma cells through single cell gel electrophoresis (comet assay). Our findings provide the first evidence that the earliest structural change induced by MTA in human melanoma cells is DNA damage after 24 h of treatment. Furthermore, this induced DNA damage could be the basis for the arrest of cells in the S-phase of the cell cycle detected in cells after MTA treatment. In accordance with our results, previous studies have shown that cell cycle arrest may occur as a result of DNA damage, in order to allow DNA repair and prevent entry into mitosis in the presence of damaged DNA [27,28].

Recent studies suggest that mitochondria could function as the principle sensor in cells, and that the release of mitochondrial factors, such as cyt c, AIF and other proteins, is the critical event governing cell fate [29,30]. Caspase-2 activation is known to be able to control the progress of apoptosis by regulating mitochondrial membrane integrity and mitochondrial potential [31]. Our study of the effect of MTA on the mitochondrial membrane potential corroborates this claim, since we found MTA induced hyperpolarisation as an early sign of apoptosis [5,32] followed by hypopolarization and mitochondrial membrane collapse. To further confirm the involvement of mitochondria in MTA-induced apoptosis, we studied the release of cyt-c and AIF from mitochondria to the cytosol. We found that MTA released cyt-c from the mitochondria to the cytosol and activates caspase-9 [33]. Furthermore, the specific caspase-9 inhibitor was the most powerful inhibitor of MTA-induced apoptosis. The release of AIF to the cytosol was further confirmed by immunocytochemistry and Western blot studies. Since AIF is a factor which produces apoptosis by acting directly on the nucleus, independent of caspase activation [34], this finding is compatible with the idea that MTA can induce both caspase-dependent and –independent apoptosis.

One of the most reproducible inducers of apoptosis is mild oxidative stress. The mechanism by means of which an oxidative stimulus can activate the caspase cascade during apoptosis has not been fully characterized; nevertheless, oxygen and its compounds is known to be able to deplete reduced glutathione or damage the cellular antioxidant defence system [35]. This leads to increased intracellular levels of ROS which are responsible for inducing DNA damage and p53 activation with subsequent apoptosis [4,28,36]. Furthermore, some proapoptotic agents, such as certain histone deacetylase inhibitors, induce caspase-independent apoptosis through the generation of ROS [37]. Based on these reports and our finding that MTA induces DNA damage, we examined the effect of MTA on the generation of ROS. MTA was found to generate a significant increase in the levels of ROS at the early stages of apoptosis, even before the caspase cascade activation. In addition, we found that pretreatment of melanoma cells with NAC significantly decreased DNA-damage, p53 expression and apoptosis in response to MTA.

It is well known that p53 is able to arrest the cell cycle, activate DNA repair proteins when DNA has sustained damage, and initiate apoptosis if the damage is irreparable [31,38]. To assess the hypothesis that MTA induces p53 expression caused by DNA damage, we studied the expression of p53 and related genes upon MTA exposure. We found p53 upregulation as a direct consequence of ROS accumulation, since pretreatment with NAC was found to prevent this altered expression. Also, knockdown of p53 functional activity by PFT-alpha resulted in a significant reduction of MTA-induced cell cycle arrest at S-phase. However, no significant reduction of cells undergoing subG0-G1 was detected when inhibiting p53 functional activity upon MTA exposure.

Subsequently, we assessed the expression of three p53-related genes which are relevant apoptosis inducers: PUMA, PIDD and Mcl-1. MTA provoked a p53-dependent expression of Mcl-1 and PIDD, together with a p53-independent PUMA regulation, since it kept an equal expression pattern next to the transient knockdown of p53. Furthermore, western blot analysis of PUMA protein did not show any relevant change upon MTA treatment, suggesting a postranscriptional regulation of PUMA and playing down the importance of PUMA in this MTA-induced cell death.

PIDD is a protein that has been shown to be positively regulated by p53 and to induce cell apoptosis in response to DNA damage [8,27,38]. This up-regulation of PIDD may result in a RAIDD and caspase 2 enhanced recruitment in order to form the PIDDosome complex, which induces the activation of caspase 2 as an initiator of the caspase cascade, as has been reported elsewhere [31,39].

Finally, we found a possible cause-effect link between p53 and Mcl-1 regulation, because transient knockdown of p53 resulted in a lower increment of Mcl-1 gene expression following MTA exposure in human melanoma cell lines. Although, similarly to PUMA, the elevated mRNA levels of Mcl-1 obtained by real-time RT-PCR did not correlate with the marked lower levels of Mcl-1 protein, despite the protein decrement appeared to be also p53-dependent. As some authors have stated, this kind of disparities are consistent with microRNA regulation of translations, where mRNA levels remains unaltered by the microRNA activity given that they exert their activity through the maturation or nuclear export process. P53 has been reported as a potentially regulator of microRNA, monitoring the expression of miRNA which are able to bind with the promoter region of Mcl-1 or miRNAs affecting the postranscriptional regulation blocking the synthesis of protein [40-42].

This MTA-induced p53-dependent regulation of Mcl-1 support the proposal of this antifolate as a potential therapeutic agent, since it has been suggested that upregulation of Mcl-1 represents an early event associated with melanocyte malignant transformation and chemotherapy resistance [43,44]. In addition, Mcl-1 is considered to be a relevant player in the induction of apoptosis in melanocytes through Apaf-1, cyt-c or Bax inhibition, and is considered to be a suitable molecular target for enhancing chemosensitivity in human melanoma [45-47].

All together these results and given the fact that degeneracy is a common event in biology, a single activation of p53 might not be essential for cell cycle progression, and apoptosis might not only depend on p53 activity but its induction seems to have a relevant role in regulating through both direct and indirect mechanisms the expression of decisive executioners of MTA-induced apoptosis.

## Conclusions

We found that MTA induced DNA damage and mitochondrial-mediated apoptosis in human melanoma cells in vitro and that the associated apoptosis was both caspase-dependent and –independent. These findings may have therapeutic relevance for the future treatment of human malignant melanoma. Our results do not shut the door to additional pathways bolstering the apoptotic stimulus likely to be important in the MTA-induced apoptosis. Future studies will be aimed at elucidating the precise p53-mediated regulation mechanisms and studying the synergistic effect of MTA with other standard chemotherapeutic agents currently in use for melanoma. Furthermore, in the light of favourable clinical experience with MTA for other malignant diseases, our results should encourage clinical trials on the effect of MTA on human metastatic melanoma.

## Abbreviations

MTA, Pemetrexed: Alimta®; NSCLC: Non Small Cell Lung Cancer; ROS: Reactive Oxygen Species; NAC: N-Acetyl-L-Cysteine; Cyt-c: Cytochrome-c; AIF: Apoptosis Inducing Factor.

## Competing interest

The authors declare that they have no competing interests.

## Author’s contribution

AB undertook the experimental procedures as well as preparing and writing the manuscript. JSM undertook the apoptosis experiments as well as preparing and writing the manuscript. AB and JSM contributed equally to this work. UA and BC undertook the gene expression assays. UA, SC and AM did the statistical analysis for the manuscript. SC, AM, AS and IR proposed the chemotherapy for the study. GLV was central in the design, coordination and manuscript editing. All authors have read and approved the final manuscript.
